# Meterological Report for December, 1879

**Published:** 1880-01

**Authors:** H. Hall

**Affiliations:** Corp’l. Signal Service U.S. A.; Atlanta, Ga.


### METEOROLOGICAL REPORT FOR DECEMBER, 1879.

| RAIN. DATE | RAIN. IN. | Mean daily Barometer. | Mean daily Thermometer | Mean daily Humidity. | Maximum Thermometer | Minimum Thermometer | Prevailing direction of Wind. | Maximum hourly velocity of Wind, (Daily.) |
|---|---|---|---|---|---|---|---|---|
| 1 | 0 | 30.384 | 40.5 | 42.3 | 49 | 30 | E. | ......... |
| 2 | 0 | 30.222 | 50.2 | 64.0 | 58 | 56 | E. | ......... |
| 3 | 90 | 30.089 | 58.2 | 81.0 | 66 | 50 | S. | ......... |
| 4 | 02 | 30.136 | 57.0 | 79.3 | 64 | 50 | S. | ......... |
| 5 | 0 | 29.957 | 54.7 | 86.7 | 59 | 51 | E. | ......... |
| 6 | 54 | 29.803 | 52.0 | 78.0 | 59 | 45 | W. | ......... |
| 7 | 0 | 29.981 | 53.5 | 57.7 | 62 | 42 | W. | ......... |
| 8 | 0 | 30.121 | 57.0 | 68.3 | 64 | 47 | S. E. | ......... |
| 9 | 0 | 30.154 | 58.7 | 80.7 | 64 | 51 | S. E. | ......... |
| 10 | 0 | 29.944 | 64.5 | 80.7 | 70 | 58 | S. E. | ......... |
| 11 | 1.57 | 29.954 | 45.2 | 80.0 | 65 | 38 | N. W. | ......... |
| 12 | 0 | 30.233 | 39.2 | 59.3 | 47 | 31 | N. W. | ......... |
| 13 | 55 | 30.313 | 37.5 | 78.0 | 43 | 34 | E. | ......... |
| 14 | 3.76 | 29.965 | 41.5 | 100.0 | 45 | 35 | E. | ......... |
| 15 | 04 | 30.170 | 45.2 | 47.6 | 50 | 40 | N. W. | ......... |
| 16 | 0 | 30.177 | 45.2 | 60.0 | 53 | 34 | N. W. | ......... |
| 17 | 0 | 30.159 | 55.7 | 60.0 | 62 | 37 | N. W. | ......... |
| 18 | 0 | 30 230 | 56.2 | 65.0 | 67 | 46 | S. | ......... |
| 19 | 0 | 30.237 | 58.0 | 54.0 | 66 | 49 | S. | ......... |
| 20 | 0 | 30.165 | 57.2 | 63.0 | 64 | 47 | S. W. | ......... |
| 21 | 0 | 30.100 | 61.0 | 85.0 | 67 | 56 | S. | ......... |
| 22 | 05 | 30.054 | 64.2 | 87.0 | 69 | 59 | S. W. | ......... |
| 23 | 01 | 30.112 | 65.2 | 86.0 | 69 | 62 | S. W. | ......... |
| 24 | 09 | 30.150 | 65.5 | 84.0 | 71 | 60 | S. | ......... |
| 25 | 29 | 30.170 | 43.7 | 81.0 | 67 | 30 | N. W. | ......... |
| 26 | 0 | 30.353 | 24.2 | 53.0 | 35 | 17 | N. W. | ......... |
| 27 | 0 | 30.304 | 38.2 | 49.0 | 48 | 22 | E. | ......... |
| 28 | 0 | 30.214 | 46.5 | 59.0 | 55 | 31 | S. E. | ......... |
| 29 | 0 | 30.236 | 52.2 | 71.0 | 60 | 41 | S. W. | ......... |
| 30 | 0 | 30.252 | 42.0 | 72.0 | 68 | 48 | S. W. | ......... |
| 31 | 04 | 30.308 | 62.5 | 79.0 | 68 | 58 | W. | ......... |
| Sums | 7.86 | 934.647 | 1592.2 | 21.916 | 18.54 | 13.55 | ......... | ......... |

Mean Monthly Barometer, 30.150.
Highest Barometer, 30.479, on the 1st.
Lowest Barometer, 29.710, on the 6th.
Monthly range of Barometer, 0.769.
Highest Temp. 71°, on 24th. Lowest Temp. 17°, on 26th.
Monthly range of Temperature, 54°.
Greatest daily range of Temperature, 37°, on the 25th.
Lowest daily range of Temperature, 7°, on the 23rd.
Mean of Maximum Temperatures, 59.
Mean of Minimum Temperatures, 43.
Mean daily range of Temperature, 16. Total rainfall, 7.86 inches.
Prevailing wind, S. W. Total movement of wind, 79.15 miles.
Maximum velocity of wind and direction, 28 miles per hour, N. W., 25th and 26th.
Number of Foggy days, 0.
Number Cloudy days on which rain fell, 7.
Number of cloudy days on which no rain fell, 5.
Total number of days on which rain fell, 12.

H. HALL, Corp'l. Signal Service U. S. A.

Atlanta, Ga., Jan. 1, 1880.

